# Gab Adapter Proteins as Therapeutic Targets for Hematologic Disease

**DOI:** 10.1155/2012/380635

**Published:** 2011-12-14

**Authors:** Sheetal Verma, Tamisha Vaughan, Kevin D. Bunting

**Affiliations:** ^1^Aflac Cancer Center of Children's Healthcare of Atlanta, Department of Pediatrics, Emory University, Atlanta, GA 30322, USA; ^2^Aflac Cancer Center and Blood Disorders Service, Division of Hematology-Oncology, Department of Pediatrics, Emory University School of Medicine, 2015 Uppergate Drive NE, ECC 444, Atlanta, GA 30322, USA

## Abstract

The Grb-2 associated binder (Gab) family of scaffolding/adaptor/docking proteins is a group of three molecules with significant roles in cytokine receptor signaling. Gabs possess structural motifs for phosphorylation-dependent receptor recruitment, Grb2 binding, and activation of downstream signaling pathways through p85 and SHP-2. In addition, Gabs participate in hematopoiesis and regulation of immune response which can be aberrantly activated in cancer and inflammation. The multifunctionality of Gab adapters might suggest that they would be too difficult to consider as candidates for “targeted” therapy. However, the one drug/one target approach is giving way to the concept of one drug/multiple target approach since few cancers are addicted to a single signaling molecule for survival and combination drug therapies can be problematic. In this paper, we cover recent findings on Gab multi-functionality, binding partners, and their role in hematological malignancy and examine the concept of Gab-targeted therapy.

## 1. Discovery and Similarities of Gab Family Members

The Gab proteins, Gab1, Gab2, and Gab3, comprise a family of scaffolding/docking molecules involved in multiple signaling pathways mediated by receptor tyrosine kinases (RTKs) and non-RTK receptors. Gab proteins integrate and amplify signals from a wide variety of sources including growth factor, cytokine, and antigen receptors, as well as cell adhesion molecules. They are subject to complex regulation by feedforward and feedback phosphorylation events as well as protein-protein interactions. Gab proteins range from 50 to 100 kDa in size [1] and were originally identified as the mammalian homologs of the *daughter of sevenless *(DOS) *Drosophila* adapter proteins [2, 3]. They also display sequence similarity to *Suppressor of Clear* 1 (*Soc*1), which was identified by genetic screen in *C. elegans *[3, 4].

Gab1 was originally identified as a binding protein for Grb-2 [5], and Gab2 was isolated by the purification of a binding partner for SHP [6]. The discovery of Gab3 was aided by a large sequencing project, and its isolation was based on sequence similarities to Gab1 and Gab2 [7]. Very recent entries at both the genomic DNA and transcript level have been recorded for Gab4 gene in both humans and chimpanzees, but this gene is not present in mice. The human Gab4 gene is located on chromosome 22q11.1 and its nucleotide sequence is most related to Gab2 [8].

**Figure 1:**
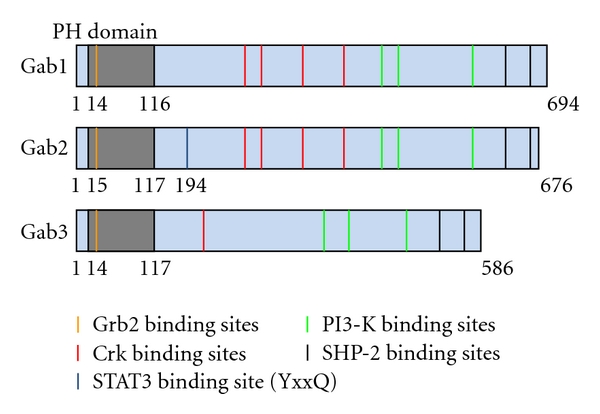
Gab structure and multiple binding sites. Gab molecules were originally identified as the mammalian homologs of the daughter of sevenless (DOS) drosophila adapter protein. Gab1, Gab2, and Gab3 share many common binding sites. All three Gabs have a PH domain in the amino-terminus that is believed to be essential for many functions including membrane localization. Gabs contain multiple binding sites and act as scaffolding molecules to support cytokine signaling. Binding sites for Grb2, Crk, PI3-K, and SHP-2 have been defined and extensively studied.

The overall sequence homology between Gab family members is about 40–50%. All Gab proteins share a similar modular structure, including a Pleckstrin homology (PH) domain at their N-terminus, proline-rich regions in the central part, and multiple phosphorylated tyrosine residues (Figure 1). The PH domain is an approximately 100 amino acid domain that binds phosphoinositides. Gab2 binds preferentially to the PI-3K product phosphatidylinositol-3,4,5-trisphosphate (PIP3), which is only found within the plasma membrane [9]. The PH domain mediates recruitment of Gab2 to phagocytic cups induced by Fc*γ*RI and is required for fibroblast growth factor-induced tyrosine phosphorylation of this docking protein. The PH domain might play an important role to localize or to concentrate Gab proteins to membrane areas where receptors are activated [2].

The phosphotyrosine domains and the proline-rich sequences (PXXP) are potential binding sites for Src homology2 (SH2) and SH3 domains, respectively. The positions of the SH2-binding tyrosine-based motifs and the SH3-binding proline-rich sequence are conserved among the Gabs. These are one of the most prominent motifs in signaling molecules due to their relevance in binding and “docking” phosphorylated tyrosine residues or directing protein-protein interaction [10, 11].

These adapter proteins serve important roles in cytokine receptor signaling by acting as a scaffold and coordinating interactions between signaling intermediates. Multiple protein binding motifs are present in many of the adapter molecules leading to multimeric complexes that may also include proteins such as CrkL [12], PLC-*γ* [13], SHIP [14], SHP-2 [15], STAT3 [16], and STAT5 [17].

## 2. Involvement in Normal and Leukemic Signaling Pathways

**Figure 2:**
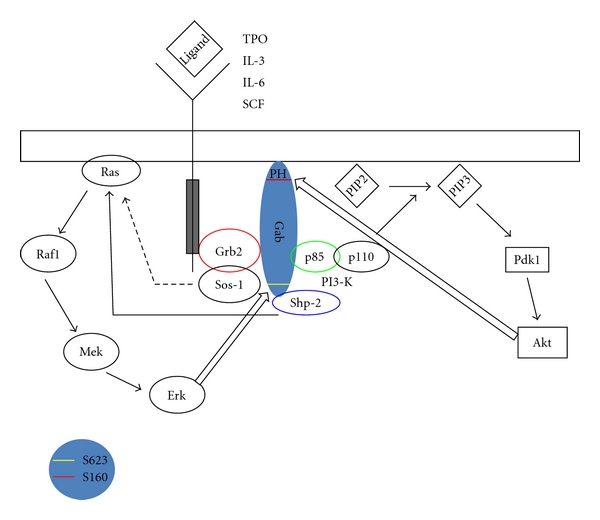
Gab2 interactions with binding partners. Diagram shows generic Gab adapter docked at the plasma membrane by the PH domain. Interactions with p85 and SHP-2 are involved in Akt and Erk activation respectively. Binding of receptor tyrosine kinases to their receptive ligands triggers the kinase activity of the cytoplasmic domain of the receptor. The receptor becomes phosphorylated on tyrosine residues. Docking proteins such as Grb2 contain SH2 domains that bind to the phosphotyrosine residues of the activated receptor. Grb2-Gab interacts through the SH3 domains and activates downstream signaling pathways, PI-3K/Akt and SHP2/Erk. Grb-2 can bind to SOS via N-terminal SH3 domain while the C-terminal SH3 domains are used for its interaction with Gab proteins. SOS-1 has been known to associate with Grb2, leading to its autophosphorylation. This complex gets translocated to the activated receptor where it then associates with Ras. As Ras gets activated it induces the downstream ERK/MAPK pathway. Alternatively, stimuli from growth factors like EGF, VEGF, and so forth, causes binding of Gab2 to Grb2. This then leads to recruitment of SHP2, that binds to phosphorylated tyrosine residues on Gab, and in turn activates ERK/MAPK signaling. Negative feedback by serine phosphorylation of Gab at S160 by Akt and S623 by Erk (block arrows) plays an important role in control of function and signaling of Gabs.

As mentioned above, one of the fundamental mechanisms for regulation of Gab-mediated signal transduction is site-specific tyrosine phosphorylation of these proteins. These molecules are involved in the phosphatidylinositol-3 kinase (PI3-K) and mitogen-associated protein kinase (MAPK) pathways and include multiple protein binding sites [18]. To further elaborate, Figure 2 illustrates how Gab2 is involved in PI-3K and MAPK pathways. These proteins are tyrosine phosphorylated following cytokine stimulation which enables interaction with a large number of partners.  Table 1 summarizes a few key receptors which are associated with hematopoiesis, some of which are also found mutated in association with hematologic malignancy. Serine phosphorylation of Gabs by downstream effectors also has been described [19, 20], which will be discussed later.

The significance of the phosphorylation sites present on Gabs not only lies in aiding its interaction with crucial binding partners, but also on how it influences activation of downstream cytokine receptors. Interaction between the protein tyrosine phosphatase Shp2 and Gab2 regulates MAPK pathway activation. Notably, mast cells and macrophages from Gab2^−/−^ mice have decreased Erk activation in response to SCF [27]. Gab2-Shp2 complex also appears to have an additional, distinct signaling role in response to other stimuli. For example, overexpression of Gab2^Y604F/Y633F^ mutant fails to bind to Shp2 and blocks IL-3-evoked gene activation [6]. Further, the Gab/Shp2 complex also positively regulates other downstream pathways. These include c-Kit-induced Rac activation [28], where Gab2 via Shp-2 transmitted signals from Kit receptor (Tyr-567) to activate the Rac/JNK pathway. This in turn is significant for mast cell development [28]. Another example of downstream regulation is the Gab2-mediated PI3K activation wherein, this activation is necessary for epidermal growth factor- (EGF-) induced DNA synthesis in rat hepatocytes [58, 59].

It is noteworthy that persistent activation of c-Kit and c-Mpl induces hematological malignancies. Activation of PI-3K by c-Kit is dependent both on the direct PI3K-binding site in c-Kit and on the phosphorylation of Gab2 [54]. The fact that c-Kit has been found mutated in numerous human malignancies, including acute myeloid leukemia, and that Gab2 is often overexpressed in acute myeloid leukemia suggests a potential role of Gab2-mediated PI3K activation in transformation [60]. TPO-induced stimulation of c-Mpl has been implicated in maintaining HSC quiescence and also in myeloproliferative disorders (MPDs) and Gabs play a role in regulating PI-3K and MAPK pathways, in c-Mpl/TPO signaling [55, 61].

## 3. Functional Role in Hematopoiesis Defined by Knockout Mice

Given their integral role in cytokine signaling, it was proposed that Gabs may play important roles in hematopoiesis. However, to date, very little is known about how multiple Gabs regulate hematopoietic cytokine signaling.

Gab1 deficiency results in embryonic lethality due to developmental defects in heart, placenta, skin, and a reduced ratio of liver to body weight at E14.5 [62, 63]. Also associated with these defects was reduced Erk activation in embryonic fibroblasts in response to stimulation with platelet-derived growth factor (PDGF), epidermal growth factor (EGF), and hepatocyte growth factor (HGF). These defects were initially observed to be similar to mice lacking expression of MET receptor, HGF, PDGF, and EGF growth factors with phenotypes such as open eye lids [64], abnormal hair follicles [65], hemorrhage and cardiac hypoplasia [66], and abnormal placenta [67–69]. Later generation of SHP-2 mutant mice revealed yet again similar defects [70] indicating an essential central role for Gab1/SHP-2 interactions in mediating growth factor activation of the Erk MAP kinase pathway. More recent conditional deletion of Gab1 led to deficient Erk signaling which allowed increased insulin receptor substrate (IRS) activation to enhance glucose tolerance and improve hepatic insulin action [71]. A role for Gab1 as an adapter protein linking gp130 signaling to the Erk pathway has also been described [39]. Gab1^−/−^ cells are defective in response to gp130 activation through IL-6 and the soluble IL-6R*α* [62].

Gab2 is tyrosine phosphorylated at any of 19 sites by several early-acting cytokine receptors such as Flt3, c-Kit, IL-3R, and c-Mpl. Gab2 contains SH2 domain binding sites and 5 PxxP sites (polyProline sites) that bind SH3 domain [6, 18, 29]. Consistent with binding of p85 and SHP-2, Gab2 activates the PI3-K and the MAPK pathways, respectively, which may participate in regulating hematopoietic cell migration functions [48]. Gab2^−/−^ mice are viable but lack allergic response [72] since Gab2 deficiency has also been shown to alter mast cell development [27] in a manner similar to STAT5-deficient mice [73]. Use of point-mutants [36, 48, 74], that deter Gab2 binding to signaling partners, could help to dissect the structure-function relationship of Gab2 *in vivo*. A recent study has established Gab2 ΔSHP2 (Y603F/Y632F) and Gab2 ΔPI3K (Y441F/Y465F/Y573F) mutant knockin mice [75], with Gab2 mutated at SHP-2 and PI3K binding sites, respectively. Further assessment of these mice has shown that the PI3K or SHP2 binding sites in Gab2 are important for mast cell degranulation and the anaphylaxis response.

Despite the normal appearance, normal BM cellularity, and normal blood counts of mice lacking Gab2 expression, Gab2^−/−^ mice show reduced colony forming ability in methylcellulose and impaired KLS (c-Kit^+^ Lin^−^ Sca-1^+^ cell surface markers denoting mouse hematopoietic stem cell) cell growth in liquid culture [76]. The defects of these cells in response to early-acting cytokines like SCF, TPO, and IL-3 suggest that Gab2 may act as an intermediate relay protein that organizes signaling complexes and amplifies receptor activation. Owing to these findings, Gab2 can be a potential target molecule for better understanding steady-state and aberrant hematology.

In contrast to Gab1 and Gab2 which have ubiquitous expression in brain, kidney, lung, heart, and ovary [5, 6, 9], Gab3 is localized to hematopoietic tissues [7, 77]. An additional difference between Gab3 and Gab1/2 is that it may not be able to interact with Crk or Crkl but has unique potential binding sites that have yet to be characterized [7]. Gab3 SH3 domains have been shown to associate with a number of Src family kinases including Src, Fyn, and Lyn [7]. Increased Gab3 expression is observed following M-CSF stimulation of myeloid and macrophage cell lines [7], and differentiation is facilitated by overexpression. However, Gab3 knockout mice do not have obvious hematopoietic phenotypes [78] and have normal macrophage numbers.

## 4. Supporting Role for Gabs in Cancer Progression

It is well established that Gab proteins promote tumor-genesis by functioning as “accomplices” of certain oncoproteins or by amplifying signaling upon their overexpression. This type of “nononcogene addiction” has been described for molecules that become essential in the setting of cancer, but they are not mutated or capable of transformation on their own. In addition to normal cytokine activation, Gab1 and Gab2 can also be activated by oncogenic tyrosine kinases, oncoproteins, and Src family kinases (summarized in Table 2). Gab3 has not yet been described to play a role in cancer signaling.

The study of Gab1 in Met signaling and cancer has been researched in recent years. Overexpression of Gab1 promotes cell cycle progression when Met is expressed at suboptimal levels. For this response, it is required for Gab1 to possess an intact Met-binding motif, the PH domain, and the binding sites for PI3-K and SHP-2. In this model, Gab1 sufficiently promoted transformation and proliferation of fibroblasts [93]. A role for Gab1/Shp-2 interaction in growth and transformation of NIH 3T3 fibroblasts has also been reported [94], although this has not yet been validated *in vivo* for disease induction. It was shown in recent studies that Gab1 expression increased cell motility and adhesion of myeloid 32D cells in a hepatocyte growth factor (HGF) stimulated setting. In this setting, Gab1 was also seen to up regulate *GATA-2, *which has been implicated in CML and could be a key player in malignant transformation [95].

Involvement of Gab2 in leukemogenesis was highlighted when myeloid progenitors from Gab2-deficient mice were found to be resistant to transformation by Bcr-Abl [87]. Phosphorylation of Y177 within the Bcr moiety leads to recruitment of the Grb2/Gab2 complex and triggers downstream signaling *via *SHP2 and PI-3K, which is crucial for enhanced proliferation and survival. Similarly, the oncogenic Bcr-FGFR1 fusion protein, which is also the product of a chromosomal translocation, drives the tyrosine phosphorylation of Gab2 in murine bone marrow cells and their malignant transformation through phospho-Y177 mediated Grb2 association [96]. Another kinase implicated as a key component of the Bcr-Abl signaling network is Jak2 that in turn activates Lyn leading to Gab2 phosphorylation. These findings highlight the role of Gab2 phosphorylation in driving chronic myeloid leukemia (CML) [97]. After the pivotal role of Gab2 in Bcr-Abl-mediated transformation had been established, its involvement in the pathogenesis of several other leukemias was discovered. The oncogenic fusion kinases Tel-Abl and Tel-Jak2 engage Gab2 in a similar manner to Bcr-Abl [98, 99]. Likewise, it was seen that introduction of a germline gain-of-function SHP2 mutation, D61G/+, induced MPD by aberrant activation of HSCs and the disease phenotype was improved in the Ptpn11(D61G/+)/Gab2(−/−) double mutant mice [100]. This further illustrates that interactions between Gab2 and partners like SHP2 is critical for development of MPD, *in vivo*. However, the relative contribution of the Gab2/SHP2 interaction for Erk activation versus the reciprocal inactivation of STAT5 is highly complex and difficult to discern [21, 90, 91, 100]. Both Erk and STAT5 can drive myeloproliferation, and the degree to which they cooperate in normal and leukemic hematopoiesis is not well defined.

It should be pointed out that although the focus of this review is on Gabs as interaction partners of oncoproteins involved in the transformation of hematopoietic cells, the most thoroughly described roles have been in solid tumors. Gab2 amplification has been seen in nonhematopoietic cancers such as breast cancer cell lines [79]. Gab2-mediated activation of the Shp2/Erk signaling pathway is important for the proliferation of mammary cells. Amplification of Gab2-containing region has been reported in 10–15% of human breast tumors [80]. Further supporting this is the *in vitro* study whereby overexpression of Gab2 in human MCF-10A cells using a retroviral vector approach [81] gave similar results. Furthermore, deletion of Gab2 delays migration of mouse mammary tumors generated using breast cancer cell lines and this defect can be fully restored by reintroduction of a plasmid expressing Gab2 [82]. A particular role for Gab2 and Akt activity has been shown following E2F1 hyperactivation in p27-deficient cells leading to enhanced cell migration and invasion [101]. A recent study in MCF-10A epithelial cells show Gab2 overexpression enhances cell migration and reduces formation of epithelial colonies. Further, modulation of focal adhesions by Gab2 was dependent on Shp-2 binding sites. Shp-2 binding defect mutant restored normal cell spreading. In contrast, the Shp-2 affinity mutant promoted Vav2 phosphorylation and recruitment of some important RhoA family regulators leading to increased cell motility [80, 102, 103].

## 5. Regulation by Posttranslational Modifications

The key to figuring out the role of Gabs in hematological disease is to understand their role in signaling cascades. It is crucial to visualize these as intertwined loops. Firstly, phosphorylation of a particular residue might affect the phosphorylation of a nearby residue in either a positive or antagonistic fashion, due to phosphorylation-induced changes in protein conformation. Secondly, phosphorylation-induced conformational changes may alter the accessibility of key regions, such as the PH domain.

**Figure 3:**
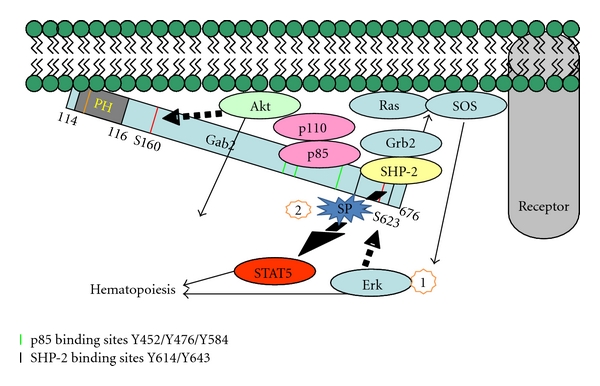
Potential of stapled peptides in therapeutic intervention. Step 1 illustrates how Erk phosphorylates Gab2 on a consensus phosphorylation site at serine 623, a residue located between tyrosine 614 and tyrosine 643, which are responsible for Gab2/Src homology 2 domain-containing tyrosine phosphatase- (SHP-) 2 interaction. As is reported in the text, this is part of a negative feedback loop. Hypothetically a stapled peptide (SP), a biosynthetic molecule binding to a directed region on Gab2, could be introduced in Step 2 to block the negative regulation by Erk to keep SHP-2 active and STAT5 inactive. Alternatively, it may be necessary to block SHP-2 binding to Gab2 depending on the disease entity. This figure elaborates how targeted therapy might provide new direction into understanding the interactions between adapters, like Gabs, and their partners, and which could ultimately be applied as leukemia therapy.

Negative feedback regulation of Gab2 can be achieved by serine phosphorylation at sites near the PH domain (Akt) or near the SHP-2 binding domain (Erk). Akt can constitutively associate with Gab2, phosphorylate it on a consensus phosphorylation site (Ser159) and inhibit Gab2 tyrosine phosphorylation [20]. Mutation of S159A (corresponding mutation in mouse is S160) resulted in increased tyrosine phosphorylation of Gab2 and the Gab2^S159A^ mutant displayed transforming properties in fibroblasts and prolonged signaling through the PI-3K/Akt pathway. This might impact downstream STAT5 activity, as it has been shown that constitutively active STAT5 forms a complex with the p85 subunit of the PI3-K and Gab2 in leukemic bone marrow cells, resulting in the activation of Akt [17]. Gab2 is also regulated by Erk-mediated negative feedback phosphorylation, wherein the identified S623 is the site of phosphorylation of Gab2 by Erk. The Gab2/Shp2 interaction is enhanced by S623A mutation. This is in turn expected to strongly inhibit downstream STAT5 [19]. Thus, it will be of relevance to study the balance between positive and negative signaling through both *in vitro* models and further complement it by *in vivo* analysis. Negative feedback is deemed critical in shifting Gab2 signaling from Erk and Akt to STAT5. This mechanism may be particularly relevant during conditions of hematopoietic stress such as recovery from myelosuppression or leukemic hematopoiesis. These conditions are more likely to increase Gab2-mediated Erk and Akt signaling, while inhibiting STAT5, whereas blockage or complete deficiency of Gab2 might be expected to have a reverse effect (Figure 3).

## 6. Targeted Therapy of Gabs?

Since Gabs bind many common receptors and are much less studied in hematopoiesis, it is pivotal to understand the role of binding to their partners and how it impacts oncogenic tyrosine kinase signaling. Due to the fact that all three family members are potential players in various signaling pathways, it will be interesting to see the shift in approach for targeting the Gab family. As discussed earlier, total knockdown of these proteins has varied physiological and phenotypic impacts. But, it cannot be emphasized enough that the Gab family members are also key regulators/enhancers of other oncogenic proteins and could compensate for the deficit of a family member.

Synthetic peptides that specifically bind to directed targets offer an approach for the modulation of receptor signaling and subsequent gene expression. Stabilized *α*-helix, SAH, is a stapled peptide to p53 that has been shown to prevent p53-MDM4 binding, enabling activation of p53 response and tumor growth suppression both *in vitro* and *in vivo* models [104]. Recently, engineered photoreactive stapled BH3 peptide helices covalently trap both static and dynamic protein interactors, and enable rapid identification of interaction sites, providing a new scope for targeted drug design for the BCL-2 family targets [105]. Visualizing potential sites on Gabs for similar stapled peptides can provide fascinating insights into protein-binding disruption. These disruptions could also uncover the role of binding partners in disease regulation (Figure 3). In some ways these directed peptides could be more practical as targeted therapy, since they would not silence the entire molecule, like siRNA targeting, but rather, could disturb binding and interactions of the target with regulators. However, these interactions are complex and as mentioned earlier both Erk and STAT5 are important for leukemogenesis, so it remains unclear whether promoting Gab2/SHP2 interaction to reduce STAT5 activation would be advantageous. The disadvantage of such a targeted therapy approach would be increased Erk activation. The therapeutic potential could depend on the particular upstream activating mutation and the stage of disease development. The use of new knockin animal models expressing mutant Gabs would be needed to address these important issues.

An alternative approach to targeting the specific effectors of Gab functional interactions would be to target the entire molecule as a whole. The risk of this approach is that it would be predicted to have widespread effects on Erk and AKT activation, as well as loss of potential negative regulatory functions. The total Gab targeting approach is also complicated by the potential redundancy of Gab1, Gab2, and Gab3 for specific Gab signaling functions. Only through rational dissection of Gab structure-function as related to disease progression will targeted therapy for Gabs become more rationally guided. It is appealing to consider adapter proteins as drug targets from the respect that they have the ability to impact upon multiple key oncogenic signals which may be cooperative. This “killing a flock of birds with one stone” may be optimal over even “killing two birds with one stone,” and only adapter proteins permit widespread impact on receptor-signaling molecule interactions. Therefore, Gabs are of interest in the field of targeted therapeutics and their complete deletion shows therapeutic potential in mouse models; however, drug development for these targets will require moving forward with caution and greater understanding of structure-function relationships.

## Figures and Tables

**Table 1:** Gabs are associated with multiple common hematopoietic receptors, RTK: receptor tyrosine kinase.

Receptor	Ligand	Cell type	Phosphorylated			References
			Gab1	Gab2	Gab3	
RTK						
Flt3	Flt3L	BaF3/Flt3, THP-1, RS4; 11	Yes	Yes	Yes	[7, 21]
Fms	M-CSF	FDFms, 32D-Fms, EML-Fms, BAC1.2F5, 32D, BMDM	NR	Yes	Yes	[6, 7, 22–26]
Kit	SCF	FDFms, MO7E, MC9, BMMC	Yes	Yes	NR	[23, 27–30]
Non-RTK						
EPO receptor	EPO	BaF3, UT-7, HCD-57, primary erythroid cells, R1, Namalwa, FDC-P1	Yes	Yes	NR	[13, 31–34]
G-CSF receptor	G-CSF	BaF3, DT40	NR	Yes	NR	[35]
GM-CSF receptor	GM-CSF	FDFms, BaF3, UT-7	Yes	Yes	NR	[23, 36–38]
gp130	IL-6, LIF	HepG2, BAF-B03, T47D, MM.1S, cardiomyocytes	Yes	Yes	NR	[29, 39–43]
IL-15 receptor	IL-15	T cells, MyLa2059	NR	Yes	NR	[44, 45]
IL-2 receptor	IL-2	Kit225, KT-3, T-cell blasts, T cells, NK3.3, MyLa2059	NR	Yes	NR	[18, 19, 29, 45–47]
IL-3 receptor	IL-3	BaF3, BAF-B03, primary hematopoietic cells, NIH 3T3	Yes	Yes	Yes	[29, 48–53]
Mpl	TPO	TF-1, UT-7, BaF3, primary megakaryocyte progenitors	Yes	Yes	NR	[54–57]

NR: not reported.

**Table 2:** Gab1 and Gab2 are activated by kinases, oncoproteins, and other adaptors in cancer cells.

Cancer	Gab activator	Gab1	Gab2
Breast [79–83]	Amplification, ErbB2, Src	Yes	Yes
Glioblastoma [84]	Met	Yes	No
Thyroid [85]	Ret	Yes	No
Gastric [86]	Amplification	No	Yes
Myeloma [40]	Hck	Yes	Yes
Chronic myelogenous leukemia [53, 87]	Bcr/Abl	No	Yes
Friend virus erythroblastosis [16, 88]	Sf-Stk	No	Yes
Anaplastic large cell lymphoma [89]	NPM-Alk	No	Yes
Acute myeloid leukemia [90, 91]	Flt3-ITD	?	Yes
Myeloproliferative disease [92]	JAK2^V617F^	?	Yes
